# The Question of the Surgical Treatment of Ileus

**Published:** 1890-07

**Authors:** E. Poppert


### THE QUESTION OF THE SURGICAL TREATMENT OF ILEUS.

---

E. Poppert. Archiv für Klin. Chirug. Band XXXIX. Translated from Centralblatt für Chirurgie, by M. B. Hutchins, M. D.

---

In addition to a thoroughly detailed operation of his own, the author mentions the surgical treatment of intestinal obstructions. The writer's case was as follows: "A man of 27 became suddenly ill with symptoms of a subacute intestinal occlusion, vomiting, constipation and meteorism. Received at the clinic on the fifth day of his illness, he at first declined the suggested laparotomy, and was only forced to agree to an operation two days later through marked aggravation of his symptoms. Then owing to his condition the intended laparotomy was abandoned and only an excrement-fistula was formed. After this, recovery was extraordinarily rapid. As the fistula closed in fourteen days, however, the symptoms of obstruction recurred five days later, rendering necessary the re-opening of the closed fistula. Patient again rapidly recovered. Two weeks later a third attack occurred, this time with symptoms of a wholly acute intestinal obstruction. At the laparotomy undertaken thirteen days after beginning of the attack, an acute peritonitis, limited to the lower portion of the abdominal cavity, was observed, but a "Meckel's diverticulum, adherent on its free end, which had produced constriction of an intestinal loop, was the cause of the obstruction. Double ligation of this and its excison between ligatures removed all appearance of constriction; and the patient recovered despite the pre-existing peritonitis.

According to the writer, there had at first existed a form of inner obstruction, subacute in course, but in the third attack the complication of symptoms gave evidence of an acute, elastic, intestinal incarceration; and while the formation of the "excrement" fistula sufficed in the two first attacks to remove the symptoms of obstruction, the third attack occurred despite an open and well functionating fistula. The severe consequences of this attack could only be averted by the division or loosening of the constricting band. Consequently the case proves, as the writer says, with the clearness of an exact experiment, the superiority of laparotomy to enterotomy, since the latter, after fulfilling its indication, cannot prevent recurrent attacks.

He desires that the surgical treatment shall discriminate between acutely occurring intestinal obstruction and the chronic or subacute form. He would have the choice of operation depend upon the severity of the clinical course. The more acute and severe this course the less hope of result, seems to him, offered by enterotomy. Without reference to diagnosing the nature and seat of the obstruction, he favors the immediate performance of laparotomy in entirely acute cases with severe symptoms. The presence of symptoms of collapse need not be considered a contraindication. In subacute obstructions either laparotomy or enterotomy may be performed. In very weak patients, and also where the laparotomy would be rendered difficult through meteorism and the absence of a sure diagnosis of the obstruction and its position, enterotomy may be considered the more fit operation.

As concerns the chronic cases, obstructions dependent upon fæcal accumulation, the writer believes that forming a fistula is the proper procedure, because it can certainly remove the *danger* of obstruction, supposing that impairment of intestinal peristalsis has not yet occurred. In these cases, however, after a time, the laparotomy shall remove the peculiar cause of obstruction.

For the rest, the writer believes that the simultaneous presence of peritonitis can to-day no longer be held as a contraindication for surgical treatment.

In conclusion the author reported a case of ileus in which, even to death, no vomiting was noticed; and said finally that the presence of albumen in the urine of these cases must be held as an inconstant symptom.

